# Image Transmission over LoRa Networks: Challenges, Innovations, and Practical Solutions

**DOI:** 10.3390/jimaging12090442

**Published:** 2026-09-14

**Authors:** Viacheslav Shkuratskyy, Aminu Bello Usman, Hamidreza Bagheri, Sam Hill

**Affiliations:** Computer Science, York St John University, York YO31 7EX, UK; h.bagheri@yorksj.ac.uk (H.B.); s.hill@yorksj.ac.uk (S.H.)

**Keywords:** LoRa, image transmission, sustainable IoT

## Abstract

The Internet of Things has increasingly enabled advancing real-time environmental monitoring through the integration of Low-Power Wide-Area Networks. Among these technologies, LoRa (Long Range) has emerged as a prominent communication technology due to its combination of long-range communication, low energy consumption, and affordability, making it particularly suitable for remote and infrastructure-limited environments. Its adaptability is further enhanced through the use of open-source hardware, renewable energy sources, and intelligent algorithms. Despite LoRa’s limitations in bandwidth and data rate, recent innovations enabled increasingly data-intensive applications, including image transmission. This review critically examines recent advances in image transmission over LoRa networks, synthesising approaches across four interconnected strategies: image compression, packetisation and reliability, communication optimisation, and application-specific techniques. The analysis evaluates trade-offs among image size, transmission latency, energy consumption, coverage, and reconstructed image quality. These considerations are particularly relevant for environmental sensing applications, including water quality assessment, air pollution monitoring, wildlife tracking, and underground mining. This review synthesises recent advances in LoRa-based environmental and visual sensing and highlights persistent challenges, including duty-cycle restrictions, limited throughput, and energy constraints, that must be addressed for broader adoption in data-intensive sensing applications. By analysing current strategies and proposing future directions, including adaptive encoding, lightweight encryption, and energy-aware scheduling, the review demonstrates the potential of LoRa to play an increasingly important role in enabling sustainable, scalable, and accessible Internet of Things solutions across diverse environmental settings.

## 1. Introduction

The Internet of Things (IoT) has transformed the monitoring and interaction of physical environments, enabling real-time data collection across diverse environments. Low power wide area networks (LPWANs) have been central to enabling long-range, energy-efficient IoT connectivity, which provide long-range communication with low energy consumption, an essential feature for devices deployed in remote or hard-to-reach areas. Among the various LPWAN technologies, including Sigfox and NB-IoT, LoRa has gained widespread adoption due to its balance of affordability, flexibility, and scalability.

LoRa has been investigated and deployed across a range of environmental monitoring applications, where its long-range, low-power characteristics are especially valuable. Studies have reported LoRa-based systems’ effectiveness in monitoring water quality [1], air pollution [2], radiation levels [3], wildlife movement [4], and underground mining conditions [5]. Several implementations integrate renewable energy sources, and intelligent algorithms to enhance system autonomy and adaptability. LoRa’s ability to operate in infrastructure-scarce, mobile, or hazardous environments has made it a cornerstone technology for sustainable and scalable IoT deployments.

In addition to static sensing, LoRa has also been applied in more dynamic and complex scenarios. For example, mesh networking protocols have been developed to extend coverage in large-scale deployments, and machine learning (ML) techniques have been used to optimise base station placement and communication reliability. These innovations highlight LoRa’s growing role in supporting diverse and demanding IoT applications.

While LoRa excels in transmitting low-bandwidth data such as temperature, humidity, or gas concentrations, its limited data rate, ranging from 0.3 to 50 kbps, together with its relatively small payload capacity per packet, makes image transmission particularly challenging. For example, even a highly compressed image can require several kilobytes of data, which far exceeds LoRa’s single-packet capacity (often limited to 51–222 bytes depending on the spreading factor and region). This study focuses on LoRa because of its potential to address a wide range of IoT challenges, including its ability to transmit visual data.

LoRa’s limitations in bandwidth and data rate can make tasks like image transmission difficult. However, researchers have tried different strategies to overcome these issues. Techniques like data compression, multi-hop transmission, and packet-based error correction have demonstrated that LoRa can support even complex applications, such as visual surveillance [6], remote diagnostics [7], and environmental monitoring [8] that requires image-based data.

By examining existing techniques and innovations in this field, this study highlights how LoRa can be adapted to support complex IoT applications, overcoming its inherent limitations while maintaining its core strengths of long-range, low-power communication. With continuous improvements, LoRa is positioned to play a central role in making IoT solutions more accessible, sustainable, and impactful worldwide.

Despite recent progress, transmitting visual data over LoRa remains a complex and largely open research area. The inherent limitations of LoRa’s bandwidth, data rate, and regulatory duty cycle restrictions pose significant challenges for handling large image files, real-time video streams, or high-resolution media. These duty cycle constraints, often limiting devices to transmit only a small percentage of time within a given hour, further restrict the volume and frequency of data that can be sent. While techniques such as data compression, selective transmission, and multi-hop routing have shown promise, many aspects, such as adaptive encoding, energy-aware scheduling, and robust error handling, still require further exploration. As IoT applications increasingly demand richer data formats, continued innovation is essential to make LoRa a viable solution for visual sensing in remote and resource-constrained environments.

Although previous studies and review articles have examined LoRa technology from the perspectives of LPWAN communications, IoT deployments, environmental monitoring, and network performance [9,10,11]. A dedicated synthesis of the techniques developed specifically to enable image transmission over LoRa remains limited. Existing reviews typically focus on communication architectures, coverage, energy efficiency, and protocol-level characteristics, while only a small number of review studies have specifically addressed image transmission despite its unique technical challenges [12,13]. Although ref. [12] provides a dedicated survey of image transmission via LoRa, its coverage is primarily focused on reviewing existing image-transmission approaches and discussing their general characteristics, limitations, and challenges. Similarly, ref. [13] proposes and evaluates a communication framework for image transmission over LPWANs, with a particular focus on LoRa modulation, rather than providing a comprehensive comparison of the broader range of techniques and performance trade-offs reported in the literature. This review therefore provides a focused analysis of image transmission over LoRa networks by systematically examining the approaches proposed to overcome the constraints imposed by limited bandwidth, low data rates, restricted payload sizes, and duty-cycle regulations. In contrast to existing reviews, the literature is analysed and compared from the perspectives of image compression, packetisation strategies, reliability mechanisms, transmission latency, energy consumption, communication range, and reconstructed image quality. The review further relates these techniques to key LoRa communication parameters and practical deployment constraints, including spreading factor selection, payload limitations, duty-cycle restrictions, and multi-hop communication. By synthesising the reported advantages, limitations, and trade-offs of the reviewed approaches, this work provides an integrated perspective on the current state of LoRa-based image transmission, identifies remaining research gaps, and highlights promising directions for the development of more reliable, adaptive, and energy-efficient visual IoT systems.

## 2. Review Methodology

### 2.1. Literature Search Strategy

A structured literature search was conducted to identify research studies addressing image transmission over LoRa and related LPWAN technologies. The objective of the search was to identify studies investigating the technical challenges and proposed solutions associated with transmitting image data over bandwidth-constrained LoRa networks. The literature search was performed using major scientific databases and publisher repositories, including IEEE Xplore, ScienceDirect, SpringerLink, MDPI, and Google Scholar. The search covered publications published between 2015 and present year.

The search strategy combined keywords relating to LoRa communication and image transmission. Representative search terms included: “LoRa”, “LoRaWAN”, “LoRa image transmission”, “Image transmission over LoRa”, “Image communication LoRa”, “Visual sensor network LoRa”, “LoRa image compression”, “Wireless image transmission”, “LoRa multimedia”, “LoRa-based image transmission”. Boolean operators such as “AND” and “OR” were used, where supported, to combine search terms and refine search results.

The search was designed to identify studies covering image compression, packetisation, transmission reliability, retransmission mechanisms, data-rate optimisation, multi-hop communication, energy efficiency, image-quality assessment, and other techniques relevant to image-based communication over LoRa networks.

### 2.2. Screening and Study Selection

Following the literature search, duplicate records were removed. The remaining studies were screened through title and abstract review to assess their relevance to image transmission over LoRa networks.

Potentially relevant studies subsequently underwent full-text assessment to determine eligibility for inclusion. Additional publications identified through the reference lists of selected studies were also evaluated using the same inclusion and exclusion criteria.

Priority was given to studies reporting practical image-transmission implementations, including those detailing compression techniques, packetisation methods, retransmission mechanisms, transmission reliability, energy consumption, communication range, latency, and image reconstruction quality.

### 2.3. Quality Assessment

To improve the consistency of the review, the included studies were assessed using a structured quality-evaluation framework developed specifically for this work. The assessment considered whether each study clearly reported:

- The proposed image-transmission or communication method;
- Image characteristics and compression procedures;
- Relevant LoRa communication parameters;
- Experimental or simulation setup;
- Performance metrics used for evaluation; and
- Sufficient results to support the reported conclusions.

Studies were not excluded solely because individual parameters were unavailable. Instead, missing information was explicitly recorded and considered during the comparative analysis of the reviewed literature.

### 2.4. Inclusion and Exclusion Criteria

Studies were included if they: (i) investigated LoRa or LoRaWAN communication, with particular relevance to image or visual-data transmission; (ii) examined techniques applicable to image transmission, including compression, packetisation, reliability, energy efficiency, communication range, latency, or image reconstruction; (iii) reported sufficient methodological or experimental information to support the review analysis; and (iv) were published in English between 2015 and the final literature search date. Studies were excluded if they were duplicates, were not available in full text, did not involve LoRa or a directly relevant LPWAN technology, or had no meaningful relevance to image transmission or the communication constraints associated with it. General LoRa environmental-monitoring studies based on sensor telemetry were retained only as contextual literature and were not treated as core image-transmission studies.

## 3. Overview of Wireless Communication Protocols for IoT Connectivity

Wireless communication protocols are fundamental to IoT connectivity, with protocol selection depending on application requirements, communication range, energy consumption, data rate, latency, and reliability. Several wireless communication technologies are commonly used for IoT connectivity. Bluetooth is primarily intended for short-range communication and is particularly suitable for wearable devices, smartphones, and local sensor applications. Bluetooth Low Energy (BLE) provides reduced power consumption compared with conventional Bluetooth and supports data rates of up to approximately 2 Mbps, depending on the Bluetooth version and operating mode [14,15,16,17,18,19]. Its widespread support across consumer devices provides strong interoperability; however, its relatively short communication range can limit its suitability for geographically distributed IoT deployments.

Zigbee is based on IEEE 802.15.4 [20] and is designed for low-power, low-data-rate applications. It commonly operates in the 2.4 GHz band and supports data rates up to 250 kbps [21]. Its mesh topology allows devices to relay data through neighbouring nodes, extending network coverage and improving resilience. Zigbee is therefore well suited to applications such as smart buildings, lighting, environmental sensing, and industrial automation. However, operation in the 2.4 GHz band can result in interference from other widely used wireless technologies, including Wi-Fi and Bluetooth.

Z-Wave is another low-power mesh technology, primarily associated with residential and building automation. Unlike Zigbee, Z-Wave operates in regional sub-GHz frequency bands, such as approximately 868 MHz in Europe and 908 MHz in the United States [22,23]. The lower operating frequency can provide improved propagation through walls and reduced exposure to congestion in the 2.4 GHz band. Z-Wave is commonly used for applications such as smart locks, security sensors, HVAC control, and alarms. Nevertheless, its relatively limited data rate, constrained network size, and proprietary ecosystem can restrict its applicability outside building automation.

For applications requiring wider geographical coverage than short-range technologies, LPWAN technologies provide an alternative to short-range protocols. Sigfox uses ultra-narrowband transmission in sub-GHz unlicensed spectrum and is designed for the transmission of small amounts of data over long distances. It supports very low data rates, with uplink payloads typically limited to 12 bytes, making it suitable for applications such as environmental monitoring, agriculture, asset tracking, and remote sensing [24,25,26]. Its principal advantages are low device complexity, long communication range, and low power consumption. However, its restricted payload size, limited downlink capability, dependence on network operator infrastructure, and regional availability constrain its suitability for applications requiring frequent or bidirectional communication.

Narrowband-IoT (NB-IoT) is a 3GPP-standardised LPWAN technology that operates in licensed cellular spectrum and can use existing cellular network infrastructure [27,28,29,30]. It provides substantially greater coverage and more reliable connectivity than short-range technologies while supporting higher data rates than ultra-narrowband systems such as Sigfox. NB-IoT is particularly suitable for applications such as smart metering, utility monitoring, smart-city infrastructure, and distributed industrial sensing. Power-saving mechanisms, including Power Saving Mode (PSM) and extended Discontinuous Reception (eDRX), can support long battery lifetimes for appropriately configured devices. However, NB-IoT depends on cellular network availability and typically requires operator provisioning, making it less suitable for deployments without adequate cellular coverage or where independent network operation is required. Table 1 summarises the principal characteristics of these technologies. The comparison focuses on the parameters most relevant to IoT communication technology selection rather than on detailed protocol procedures.

The comparison shows that the suitability of a wireless communication technology for IoT depends on the characteristics of the intended application. Bluetooth, Zigbee, and Z-Wave are well suited to short-range and building-scale applications, but their coverage characteristics limit their applicability to geographically distributed deployments. Sigfox provides long-range, low-power connectivity with a very low data rate and restricted payload size, making it more appropriate for small and infrequent sensor messages than for image transmission. In addition, Sigfox generally relies on operator-managed network infrastructure and subscription-based connectivity, which can introduce additional deployment and operational constraints. NB-IoT provides wider coverage and higher data rates through cellular infrastructure, but its reliance on mobile network operators, SIM-based provisioning, and network availability can reduce deployment flexibility in remote or infrastructure-limited environments.

In contrast, LoRa provides long-range, low-power communication in unlicensed spectrum and can be deployed without an inherent requirement for a cellular connectivity subscription. This deployment flexibility is particularly relevant to remote IoT applications and experimental systems involving the transmission of multiple packets to reconstruct an image. Nevertheless, LoRa’s limited bandwidth, data rate, and packet size present significant challenges for image transmission. These characteristics motivate the use of LoRa as the communication technology with subsequent sections focusing on the techniques used to enable and evaluate image transmission over a constrained long-range wireless link.

## 4. LoRa Technology for Low-Power Wide-Area IoT Networks

### 4.1. Introduction to LoRa Technology

LoRa is a wireless physical-layer modulation technology designed for low-power, long-range communication in Internet of Things (IoT) applications [31]. Operating on frequency bands like 868 (or 433) MHz in Europe, 915 MHz in the U.S., and 470–510 MHz in China, it ensures region-specific parameters for efficient communication. LoRa-based systems can support a wide range of IoT deployments, while network scalability depends on the higher-layer communication protocol and network architecture used.

LoRa supports low-power operation that can enable multi-year battery life under appropriate operating conditions [32]. However, actual battery lifetime depends on factors including transmission frequency, payload size, radio configuration, network conditions, sleep intervals, and battery capacity. The use of unlicensed spectrum and the possibility of deploying private network infrastructure can also reduce dependence on cellular operators and make LoRa attractive for remote and large-scale IoT deployments.

### 4.2. LoRa Network Architecture

LoRa is a proprietary wireless modulation technique developed by Semtech [33]. It is based on Chirp Spread Spectrum (CSS), enabling robust communication over long distances while maintaining low power consumption. This makes LoRa ideal for IoT and Machine-to-Machine (M2M) applications. LoRa provides the physical-layer communication technology used by many low-power wide-area IoT systems. It supports long-range data transmission (up to 15 km in rural areas and 5 km in urban areas) and ultra-low power consumption, enabling devices to operate on battery power for up to 10 years. At the network level, LoRaWAN uses a star-of-stars architecture in which gateways relay messages between LoRa end devices and network servers [34].

#### 4.2.1. LoRaWAN Star-of-Stars Architecture, Gateways, and Long-Range Capabilities

In a LoRaWAN star-of-stars topology, end devices transmit data directly to one or more gateways without routing through intermediate nodes. These gateways act as transparent bridges, forwarding packets to a centralized Network Server over IP-based connections such as Ethernet, cellular, or satellite backhaul. This architecture reduces complexity at the device side, as devices do not need to manage mesh networking or routing tables.

The long-range capability of LoRa stems from its combination of high link budget, narrow bandwidth operation, and robust modulation scheme. In rural deployments, communication ranges of up to 15–20 km are achievable under line-of-sight conditions, while urban deployments typically cover 2–5 km due to obstructions and interference. LoRaWAN gateways can support communication with multiple end devices, enabling scalable deployments for applications like smart metering, agriculture, and environmental monitoring.

#### 4.2.2. LoRa Modulation and Key Parameters

LoRa uses Chirp Spread Spectrum (CSS) modulation, where information is encoded in frequency sweeps (“chirps”) across the channel bandwidth. This approach offers strong resistance to interference and multipath fading, making it suitable for low-power devices communicating over long distances. Several adjustable parameters determine LoRa’s performance, trade-offs, and suitability for different use cases:

- Spreading Factor: Determines the number of chirps per symbol;
- Bandwidth: Available options typically include 125 kHz, 250 kHz, and 500 kHz. Narrower bandwidth improves sensitivity but reduces maximum throughput;
- Code Rate: Adds Forward Error Correction bits for reliability. Lower CR values maximize throughput; higher CR values improve robustness;
- Data Rate: Derived from SF, bandwidth, and CR; represents effective payload throughput.

These parameters are adjustable at the device firmware level, allowing network designers to optimize for battery life, range, or throughput depending on application requirements.

### 4.3. Chirp Spread Spectrum

LoRa is a wireless modulation technique based on Chirp Spread Spectrum. It enables long-range communication with low power consumption, making it suitable for IoT applications. Its modulation technique spreads the signal over a wide bandwidth, enhancing robustness and resilience to interference. Its performance depends on several configurable parameters, such as spreading factor, bandwidth, and coding rate, which directly affect the maximum allowable payload size and the Time-on-Air for each transmission.

#### 4.3.1. Spreading Factor

The spreading factor (SF) determines how much the signal is “spread” over time. SF defines the number of chips per symbol. The range of SF is from 7 to 12. Higher SF increases processing gain, improving reception sensitivity and range. Lower SF reduces the time on air and energy consumption for devices closer to the gateway. CSS works by encoding information into frequency-modulated chirp signals that sweep across the channel bandwidth. This improves resilience to multipath fading, Doppler shifts, and narrowband interference.

In LoRa, “chips” are the smallest modulation elements produced by the CSS process. One symbol consists of 2^SF^ chips, so increasing SF exponentially increases the number of chips per symbol. For example:

- SF7: 27 = 128 chips per symbol;
- SF12: 212 = 4096 chips per symbol.

**Figure 1:**
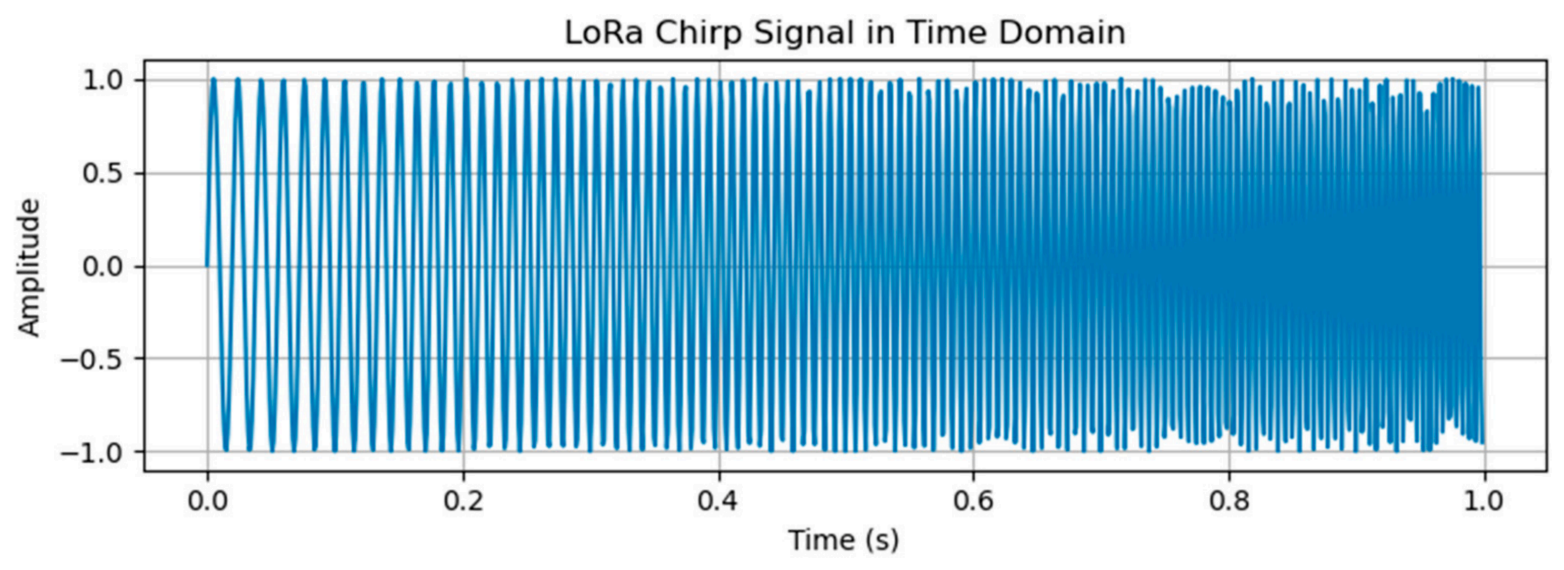
Illustrating Spreading.

The effect of SF on signal structure can be seen in Figure 1, where the instantaneous frequency of the chirp signal increases steadily over the duration of each symbol. Higher SF values result in longer symbol durations and more pronounced frequency sweeps.

This exponential increase in symbol length provides higher processing gain, the ability to detect weak signals buried in noise, but at the cost of a lower data rate and longer airtime:

- Range: Higher SF improves sensitivity (by up to ~2.5 dB per SF step), enabling longer communication distances in noisy or obstructed environments;
- Battery Life: Longer airtime means the radio stays active for longer, increasing energy consumption;
- Interference Resistance: Higher SF values are more resistant to interference and multipath fading, which is beneficial in urban and industrial deployments:
  - SF7–SF9: Used for short to medium range, higher throughput applications (e.g., smart city sensors close to a gateway);
  - SF10–SF12: Used for long-range, low data rate links (e.g., remote agriculture monitoring where range is more critical than speed).

Because LoRa uses orthogonal SFs, devices operating on different SFs can transmit on the same channel simultaneously with minimal mutual interference, increasing network capacity.

#### 4.3.2. Bandwidth

**Figure 2:**
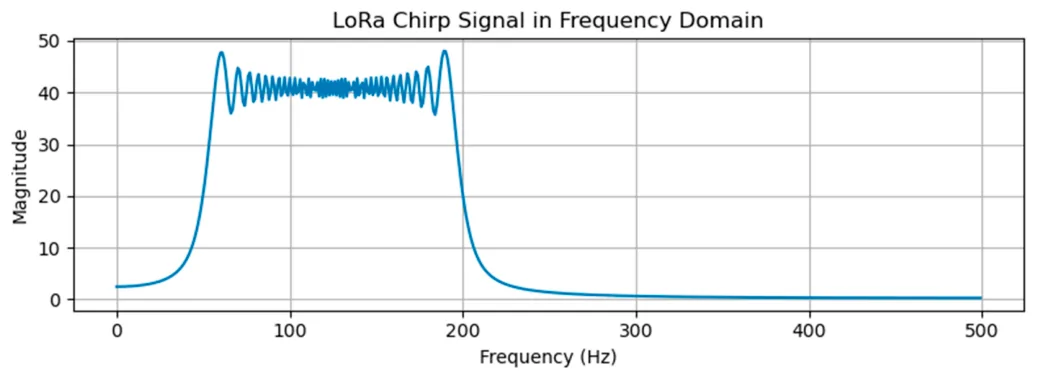
Illustrating Bandwidth.

Bandwidth is the width of the frequency channel used for communication. Common values include 125 kHz, 250 kHz, and 500 kHz. However, based on European Telecommunications Standards Institute [35] LoRa is allowed to operate at 125 kHz and 250 kHz. Wider bandwidths allow higher data rates but reduce receiver sensitivity. Narrow bandwidths improve sensitivity, which is crucial for long-distance communication. The relationship between bandwidth and channel occupancy is illustrated in Figure 2. The plot shows the spectral width of a LoRa chirp, with wider bandwidths enabling higher data rates but reducing receiver sensitivity.

In LoRa modulation, the channel bandwidth defines the range of frequencies over which the chirp signal sweeps. A wider bandwidth enables each chirp to change frequency more rapidly, allowing more symbols to be sent in a given period, increasing the data rate. However, the ability of the receiver to distinguish a weak signal from background noise is reduced as bandwidth increases. An increase in bandwidth enhances the achievable data rate, but this comes at the cost of reduced receiver sensitivity, since a wider bandwidth admits proportionally more noise power across the extended frequency range.

Conversely, a narrower bandwidth improves receiver sensitivity because the signal energy is concentrated into a smaller frequency range while less noise power is collected. The resulting increase in signal-to-noise ratio allows weaker signals to be detected at greater distances. This is why, for long-range and low-power applications, a bandwidth of 125 kHz is often preferred, despite the lower throughput. In the European ISM bands, regulatory constraints [36] limit available bandwidths to 125 kHz and 250 kHz, whereas in North America the 500 kHz option is permitted for higher throughput modes.

#### 4.3.3. Code Rate

The Code Rate (CR) is a ratio of useful bits to total transmitted bits. CR adds error correction bits for reliability. A fixed CR of 4/5 means that for every four data bits, one redundant bit is transmitted. This redundancy improves robustness but slightly reduces efficiency. LoRa supports CR values of 4/5, 4/6, 4/7, and 4/8, with higher redundancy improving reliability at the expense of throughput. CR fraction represents the total number of bits per coding block (data + redundancy) and the numerator is the number of original data bits. For example:

- CR = 4/5 → 1 parity bit for every 4 data bits (20% redundancy);
- CR = 4/6 → 2 parity bits for every 4 data bits (33% redundancy);
- CR = 4/7 → 3 parity bits for every 4 data bits (43% redundancy);
- CR = 4/8 → 4 parity bits for every 4 data bits (50% redundancy).

Increasing redundancy improves the likelihood that the receiver can correct bit errors caused by interference, fading, or weak signals. However, it comes at the cost of lower effective data rate and longer airtime, which can impact battery life and may conflict with duty cycle limits in regulated bands. In many real deployments, CR = 4/5 is chosen as a default because it balances reliability with efficiency, while higher CR values are reserved for challenging link conditions or critical data transmissions.

#### 4.3.4. Data Rate

Data rate is the rate at which data transmitted, determined by the SF, bandwidth, and CR. It represents the amount of information transmitted per second. Data rate is calculated using Equation (1).

(1) $$DR=SF\times BW\times CR\times \frac{1}{2^{SF}}$$

where:

DR—data rate (bits per second)
SF—spreading factor (7 ≤ SF ≤ 12)
BW—bandwidth (Hz)
CR—code rate (dimensionless, 4/5, 4/6, 4/7, 4/8)

Higher SF increase range but lower data rate, which reflects the exponentially longer symbol duration. Likewise, wider bandwidth increases data rate by allowing more symbols per second, but at the cost of reduced receiver sensitivity.

#### 4.3.5. Symbol Duration

The symbol duration is a key parameter in LoRa communication and is directly influenced by both the SF and bandwidth. Symbol duration represents the time required to transmit a single symbol and is calculated using the Equation (2).

(2) $$T_{S}=\frac{2^{SF}}{BW}$$

where:

$T_{S}$—symbol duration (seconds)
SF—spreading factor (7 ≤ SF ≤ 12)
BW—bandwidth (Hz)

This longer symbol duration improves sensitivity and link budget, allowing the receiver to detect weaker signals, but it also increases airtime, which lowers the achievable data rate and impacts battery life in time-sensitive applications. In practical network design, SF, bandwidth, and CR are tuned together to balance range, throughput, and energy consumption. Higher SF and lower bandwidth maximise range, whereas lower SF and higher bandwidth maximise throughput. Table 2 shows examples of how SF, bandwidth, and CR affect symbol duration and data rate.

Depending on the selected SF, bandwidth, and CR, the LoRa physical layer can operate across a wide range of raw data rates. At the lower end, configurations such as SF12, 125 kHz bandwidth, and CR = 4/8 yield data rates of approximately 180 bps, optimised for maximum range and link robustness at the expense of throughput. At the upper end, settings such as SF7, 250 kHz bandwidth, and CR = 4/5 can achieve around 11 kbps, offering higher throughput but significantly reduced range and sensitivity.

In practice, most real-world LoRa deployments operate within the low single-digit kilobits per second range (e.g. 1–5 kbps), as these configurations provide a balanced compromise between coverage, energy efficiency, and channel occupancy. It is important to note that these figures represent raw physical layer bit rates; actual application-layer throughput will be lower due to protocol overheads, duty cycle restrictions, and potential retransmissions in noisy environments.

### 4.4. LoRa Packet Formats

A fundamental component of LoRa communication is the structure of its packets, which directly affects transmission efficiency, reliability, and adaptability under various network conditions. The LoRa physical layer defines two primary packet formats: Explicit Header Mode and Implicit Header Mode, both designed to manage how transmission parameters and payload data are organised and interpreted during wireless communication [37].

#### 4.4.1. Explicit Header Mode

In Explicit Header Mode, each packet includes a complete header that specifies critical transmission parameters. Specifically, the header contains information on the payload length, the coding rate used for Forward Error Correction (FEC), and an indicator of whether a Cyclic Redundancy Check (CRC) is present. This mode allows receivers to dynamically adapt to different packet configurations, thereby enhancing reliability and flexibility, particularly in networks with varying payload sizes or coding schemes [33].

The inclusion of a header, however, introduces additional overhead, increasing airtime, which can be a limiting factor in duty-cycle-regulated environments such as the European ISM bands [36]. Despite this trade-off, Explicit Header Mode is widely adopted in scenarios requiring adaptability, mixed payload sizes, or heterogeneous device deployments. In Explicit Header Mode, a LoRa packet consists of:

- Preamble: typically, 8 symbols, but configurable from 6 to 65,535 symbols, used for synchronisation;
- Explicit Header: 3 bytes, containing the payload length, coding rate, and CRC presence flags;
- Payload: carries user application data and can range from 0 to 255 bytes depending on the SF, bandwidth, and regional regulations;
- CRC: optional field (2 bytes) providing error detection.

**Figure 3:**
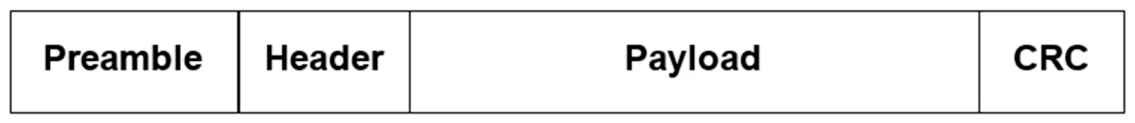
Structure of a LoRa Packet in Explicit Header Mode.

In Explicit Header Mode, the header is included and transmitted explicitly as part of the packet. The complete structure comprises the Preamble, Explicit Header, Payload, and optional CRC. Although including the header adds airtime overhead, it allows the receiver to dynamically adapt to varying payload lengths, coding rates, and CRC settings without requiring identical pre-configuration at both ends. The relative sizes and arrangements of these fields are illustrated in Figure 3, which shows the position and proportion of each component for both packet modes.

In practice, Explicit Header Mode is the default in most LoRa implementations because it ensures that any receiver tuned to the correct frequency and SF can decode the incoming packet without prior knowledge of its configuration. This flexibility is particularly valuable in shared or public LoRaWAN networks.

#### 4.4.2. Implicit Header Mode

On the contrary, Implicit Header Mode ignores the header transmission, thus reducing overhead and shortening airtime. In this mode, the receiver must be pre-configured with all necessary parameters, including payload length, coding rate, and CRC configuration. The absence of a header reduces packet size, making this format more efficient for short, fixed-size transmissions, an advantage in highly constrained networks or under strict duty cycle regulations [33].

However, the elimination of dynamic information limits flexibility and increases susceptibility to transmission errors if the receiver is not accurately configured. As such, Implicit Header Mode is primarily suited to applications with predictable traffic patterns and consistent packet structures. In Implicit Header Mode, a LoRa packet consists of:

- Preamble: typically, 8 symbols, but adjustable in the same way as Explicit Header Mode;
- Payload: carries a fixed-length block of user data agreed in advance by both transmitter and receiver, 0–255 bytes depending on configuration.

**Figure 4:**
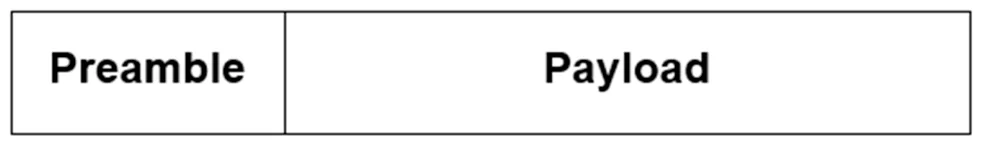
Structure of a LoRa Packet in Implicit Header Mode.

This mode omits the header entirely, reducing airtime and overhead but requiring both ends to be pre-configured with the same payload length, coding rate, and other parameters. The absence of a header makes this format more efficient for short, predictable transmissions, although it sacrifices flexibility compared with Explicit Header Mode. The relative positions and proportions of the Preamble and Payload fields in this configuration are illustrated in Figure 4, which compares the packet structures of both header modes.

Typical examples include point-to-point sensor links, where both ends of the connection are fixed in configuration, and repetitive beacon messages where every packet is identical in length and coding.

#### 4.4.3. Comparison and Use Cases

It is important to note that, at the physical layer, LoRa technology supports only these two packet formats: Explicit and Implicit Header Modes. Higher-layer protocols, such as LoRaWAN, introduce more complex frame structures including Join Request/Accept messages and Uplink/Downlink frames with MAC-layer commands. However, these operate independently of the underlying physical packet structure [33,34,37].

Explicit Header Mode is better for variable payload sizes, heterogeneous devices, or networks requiring dynamic adaptation, whereas Implicit Header Mode is preferred for fixed-size, high-frequency transmissions where airtime efficiency is crucial. The choice between the two modes is a trade-off between adaptability and efficiency: Explicit Header Mode maximises interoperability, while Implicit Header Mode minimises airtime.

### 4.5. Duty Cycle Regulations and Transmission Constraints

LoRa devices operate in unlicensed ISM bands, which are subject to regulatory constraints designed to ensure fair spectrum sharing and minimize interference between different wireless systems. One of the most significant of these constraints is the duty cycle limitation: the proportion of time a device is allowed to transmit on a given frequency channel. These restrictions vary by region and frequency band, and they directly influence application design, packet scheduling, and achievable throughput. Understanding these rules is essential for deploying compliant and efficient LoRa-based IoT networks, especially in Europe where ETSI standards are strictly enforced.

#### 4.5.1. ETSI Regulations and Their Impact on Data Transmission

In Europe, the duty cycle regulations for LoRa devices are defined by the ETSI EN 300 220-2 series of standards [36] and RP002-1.0.4 Regional Parameters [38], which govern Short Range Devices operating in the frequency range of 25 MHz to 1000 MHz. These regulations ensure fair spectrum usage and minimize interference among devices.

#### 4.5.2. Frequency Bands and Duty Cycles

LoRa technology operates with duty cycle limits of 0.1%, 1%, and 10%, depending on the frequency band, which impacts the total transmission time. For instance, with a 1% duty cycle, the system can only transmit for 36 s during each transmission cycle. This restriction complicates the transmission of image data, as high bit rates are required to effectively send the data within these limited time frames. Therefore, despite LoRa has the advantages of LPWANs, transmitting image data over LoRa remains a challenging task due to these data transmission limitations.

The standard outlines specific duty cycle limitations for various sub-bands commonly used in LoRa communication (Table 3).

For a LoRa transmission with airtime operating under a duty cycle, the mandatory waiting time before the next transmission on the same sub-band is given by Equation (3).

(3) $$T_{off}=T_{air}\times \left(\frac{1}{DC}-1\right)$$

where:

$T_{off}$—waiting time (s)
$T_{air}$—transmission time (s)
DC—duty cycle

For example, under a 1% duty-cycle restriction, an image requiring a cumulative transmission airtime of 60 s would necessitate approximately 5940 s (99 min) of mandatory off-time. Consequently, the complete transfer would take about 6000 s (100 min), assuming all transmissions occur within the same duty-cycle-limited sub-band and no additional delays or retransmissions are required.

### 4.6. Theoretical Constraints of Image Transmission over LoRa Networks

The transmission of images over LoRa networks is fundamentally constrained by the low data rates, limited payload sizes, duty-cycle regulations, and susceptibility to packet loss. Although various compression and packetisation techniques can improve transmission efficiency, the feasibility of image transmission remains governed by several theoretical and practical limitations.

#### 4.6.1. Transmission Time and Image Size

A fundamental relationship affecting image transmission is the dependence of transmission time on image size and effective communication throughput. The minimum theoretical transmission time may be expressed in Equation (4).

(4) $$T_{img}=\frac{S_{img}}{R_{eff}}$$

where:

$T_{img}$—image transmission time (s)
$S_{img}$—image size (bits)
$R_{eff}$—effective data rate (bps)

The relationship demonstrates that transmission time increases linearly with image size and decreases with increasing data rate. This illustrates the strong dependence of image-transmission performance on LoRa configuration parameters such as spreading factor, bandwidth, and coding rate.

#### 4.6.2. Impact of Bit Errors and Packet Loss

Unlike conventional sensor measurements, image transmission typically requires the successful delivery of a large number of packets. Consequently, transmission reliability becomes increasingly important as image size increases. The probability of successfully reconstructing the complete image may be approximated as in Equation (5).

(5) $$P_{img}=P_{s}^{N}$$

where:

$P_{img}$—probability of complete image reconstruction
$P_{s}$—probability of successful delivery of a packet
N—number of packets

This relationship shows that even relatively small packet loss rates can significantly affect image reconstruction.

#### 4.6.3. Shannon Capacity and Practical LoRa Limitations

The theoretical maximum data rate of a communication channel is bounded by the Shannon-Hartley theorem (6).

(6) $$C=B\;{log}_{2}\;(1+SNR)$$

where:

C—channel capacity (bps)
B—channel bandwidth (Hz)
SNR—signal-to-noise ratio

This Equation (6) defines the theoretical upper communication limit, practical LoRa systems operate significantly below this capacity due to several constraints. These include SF overhead, forward error correction, protocol headers, packet acknowledgements, retransmissions, duty-cycle regulations, and channel-access limitations.

## 5. LoRa in Action: From Scalar Sensing to Image Transmission

LoRa-based IoT systems are proving essential for scalable, resilient, and intelligent environmental monitoring. Their ability to operate in remote, infrastructure-scarce, and hazardous environments makes them a cornerstone of future smart city and ecological initiatives. The fusion of LoRa with Unmanned Aerial Vehicles (UAVs), renewable energy, and AI-driven analytics signals a shift towards more autonomous and context-aware monitoring solutions. While conventional LoRa monitoring primarily communicates scalar measurements, visual sensing can provide complementary spatial and semantic information that cannot be fully captured through parameters such as temperature, pH, gas concentration, or water level alone. This creates an emerging need to integrate image sensing with low-power, long-range communication networks.

Several studies have employed LoRa to enable real-time water monitoring using energy-efficient, cost-effective systems. A distributed IoT-based system presented in [39] to measure temperature, pH, turbidity, and conductivity with high accuracy and a 2 km range. Similarly, the study [40] autonomously collected aquatic data, achieving over 95% accuracy in obstructed environments. LoRa-based networks were tested in coastal and riverine contexts as presented in [41]. In rural settings, a solar-powered LoRaWAN system was developed in [1], demonstrating over 13 h of autonomous operation with real-time cloud transmission. Furthermore, a battery-less water-level monitoring system powered by photovoltaic cells was proposed in [42]. Although these systems primarily rely on scalar measurements, water-monitoring applications can also benefit from visual information. For example, images can provide direct evidence of algae blooms, floating debris, oil contamination, sediment plumes, or changes in water-surface conditions. A water-level or turbidity sensor may indicate that an abnormal event has occurred, whereas an image can help identify its visual characteristics and spatial extent.

LoRa has also been adapted for atmospheric monitoring in both indoor and outdoor settings. Kumar and colleagues [43] detected pollutants indoors, achieving robust transmission (590 m) and high classification accuracy via ML. For outdoor radiation detection, a LoRaWAN-based network was implemented in [44]. The study [2] developed a low-cost LoRa-based air quality system for real-time feedback across various urban environments. In such applications, visual sensing can provide additional contextual information, for example, by supporting the identification of smoke, visible pollution, vegetation stress, or other environmental anomalies that may accompany changes detected by atmospheric sensors. Thus, images can complement rather than replace conventional scalar measurements.

LoRa’s ability to function in RF-hostile environments was demonstrated in [5,45], who developed gas and temperature monitoring systems for underground coal mines. The study [3] applied LoRa mesh networking to karst caves, addressing the challenges of irregular morphology and limited accessibility. These hazardous and inaccessible environments also provide important use cases for visual monitoring. While gas, temperature, and other sensors can detect hazardous conditions, images can support remote inspection of tunnels, rock falls, smoke, equipment, and structural anomalies without requiring continuous human access.

LoRa’s low power and long-range capabilities make it suitable for integration with UAVs. A system combining ground-based IoT nodes and UAV-based LoRa receivers was presented in [46] to monitor air quality in the Delhi NCR region. This approach was further extended in [47] by using UAVs equipped with LoRa gateways for emergency environmental monitoring, achieving reliable communication over 17 km and results comparable to commercial meteorological stations. The integration of UAVs is particularly relevant to visual environmental monitoring because UAVs can collect images over areas that are difficult or unsafe to access from the ground. For example, UAV imagery can support flood-extent assessment, infrastructure inspection, vegetation monitoring, and post-disaster environmental assessment, while LoRa can provide low-power communication between distributed sensing nodes and the UAV gateway.

In ecological tracking, the study [4] developed a LoRa-based solar-powered tag for gulls, using sparse GNSS updates and ML to predict location, balancing energy consumption with positional accuracy. Advancements in LoRa protocols have enhanced scalability. Wu and Liebeherr [48] introduced a mesh networking protocol enabling over 100 nodes across hundreds of square kilometres. Another study [49] demonstrated LoRa’s potential in the Amazon basin. Their hybrid protocol enabled peer-to-peer and LoRaWAN integration, addressing long-distance, infrastructure-poor challenges. Similar ecological monitoring applications can benefit from imagery for animal identification, population counting, behavioural observation, and habitat assessment. In such scenarios, visual information can complement location and environmental measurements by providing direct information about the observed species or ecosystem.

LoRa has also been applied in diverse environmental monitoring scenarios. Coastal soil monitoring using LoRa was demonstrated in [50], achieving a battery life of up to one month and enabling the detection of rapid subsurface changes. In [51], LoRa was tested on Bidong Island, Malaysia, where the system achieved a 98.18% packet delivery rate over 23 km despite harsh conditions. Furthermore, a LoRa-based system for bee colony monitoring was deployed in [52]. Although limited to short-range communication (58 m), the system reliably captured environmental–behavioural correlations, such as sound intensity and temperature during bee activity. These examples further demonstrate the suitability of LoRa for distributed and infrastructure-poor environmental monitoring, while also highlighting situations in which scalar or acoustic measurements may benefit from visual confirmation. For example, images can support environmental surveillance by verifying events detected by sensors and providing contextual information that is otherwise unavailable from individual measurements.

Common platforms used in these studies include Arduino [53], ESP32 [54], Raspberry Pi [55], and specialised modules such as Acsip S76S [56] and Heltec Cubecell [57]. Sensor suites vary depending on the application but often include temperature, humidity, gas, and pressure sensors. Solar power and supercapacitors are frequently employed to ensure autonomous, long-term operation. These systems are designed for low maintenance and high durability. For data analytics, ML models and cloud platforms [58], ref. [59] are used for real-time analysis, alerting, and predictive modelling. When cameras are added to these platforms, however, the communication requirements change substantially. Unlike temperature, humidity, gas, or pressure readings, images contain much larger payloads and therefore impose greater demands on bandwidth, transmission time, energy consumption, reliability, and latency. Consequently, environmental image transmission over LoRa requires strategies such as image compression, event-triggered acquisition, edge-based feature extraction, AI-assisted filtering, and adaptive transmission. In a practical deployment, a scalar sensor can first detect an abnormal condition and trigger a camera to capture an image, allowing visual data to be transmitted only when required. This sensor–camera–LoRa architecture can reduce unnecessary image transmissions while retaining the contextual information needed to identify and verify environmental events.

## 6. Real-World Requirements for Image Transmission over LoRa

Image transmission requirements vary substantially according to the intended application. Unlike conventional image-communication systems, many LoRa-based visual sensing applications do not require continuous high-resolution video or real-time image streaming. Instead, they often require the transmission of low-resolution or compressed still images at relatively long intervals, with the required latency determined by the application response time. Therefore, the feasibility of image transmission over LoRa should be evaluated against application-specific requirements rather than against a single universal target for resolution, frame rate, or latency.

### 6.1. Mountain Search and Rescue

Mountain search and rescue missions often occur in environments with no cellular or Wi-Fi coverage, limiting the ability to transmit information from drones or ground sensors. LPWAN technologies such as LoRa enable long-range, low-power communication across rugged terrain, supporting the transfer of essential situational data. By enabling drones or handheld devices to send small, compressed images or sensor updates, LoRa improves visibility and coordination in areas where conventional communication infrastructure is unavailable. Requirements for maintain search and rescue:

- Data size and compression: can be implemented aggressive compression
- Long range (coverage): required, area beyond cellular/Wi-Fi footprint in rugged terrain.
- Image integrity: required, all image fragments are needed
- Security: not the primary focus, range and reliability are prioritised
- Latency: faster alerts improve coordination and response.
- Power efficiency: required, equipment must operate for extended missions.
- Equipment requirements: Harsh terrain and weather demand durable hardware
- Mobility Support: required, drones and teams move constantly

### 6.2. Disaster Relief Communications

Natural disasters frequently damage or destroy traditional communication networks, making rapid deployment of alternative connectivity essential. Temporary LoRaWAN gateways provide an immediate, self-sufficient communication layer for emergency teams. These networks support low-bandwidth data exchange, such as structural assessments, environmental readings, or lightweight image snapshots, allowing responders to map damage zones, locate survivors, and maintain information flow despite the absence of Wi-Fi, cellular, or satellite systems. Requirements for disaster relief communications:

- Data size & compression: can be implemented aggressive compression.
- Long range (coverage): required, infrastructure is damaged and links must bridge wide areas quickly.
- Image integrity: required, all image fragments are needed for reliable damage and survivor assessment.
- Security: not the primary focus, speed of deployment and operability take priority.
- Latency: faster alerts improve triage, hazard mapping, and tasking of teams.
- Power efficiency: recommended, temporary nodes and gateways may be battery/solar powered.
- Equipment requirements: rugged, rapidly deployable gateways and nodes for post-disaster conditions.
- Mobility Support: recommended, responders and UAVs move through the zone continuously.

### 6.3. Wildlife Tracking in Remote Areas

Wildlife monitoring in remote habitats typically relies on GPS sensors and radio tags, but these environments often lack access to GSM or broadband networks. LoRa’s long-range communication capabilities allow researchers to collect behavioural and environmental data from animals across large geographic areas without frequent battery replacement. Integrating lightweight visual data, such as low-resolution camera trap images, can enhance the understanding of migration patterns, predation risks, and habitat conditions while maintaining low energy consumption. Requirements for wildlife tracking in remote areas:

- Data size & compression: can be implemented aggressive compression.
- Long Range (coverage): required, animals roam in GSM-free environments over large areas.
- Image Integrity: recommended, lightweight FEC/reassembly helps with intermittent coverage.
- Security: not the primary focus, unless protection is required in anti-poaching scenarios.
- Latency: optional, most data are non-urgent; exceptions for event-triggered alerts.
- Power efficiency: required, tags/camera traps must operate for long periods without maintenance.
- Equipment requirements: weatherproof, temperature- and moisture-resistant enclosures.
- Mobility Support: optional, opportunistic uploads when animals come into coverage.

### 6.4. Smart Agriculture (Large Farms)

Large agricultural operations deploy distributed sensor networks to monitor soil conditions, microclimate, irrigation performance, and crop health. These deployments often span kilometres, surpassing the practical limits of Wi-Fi or Bluetooth. LoRa provides a cost-effective solution for long-range, low-power data collection. When combined with drone-assisted imaging or remote field cameras, LPWAN communication can support periodic transmission of small visual indicators for early detection of crop stress, pests, or irrigation failures. Requirements for smart agriculture:

- Data size & compression: can be implemented aggressive compression.
- Long range (coverage): required, fields span kilometres beyond Wi-Fi/BLE limits.
- Image integrity: recommended, but not crucial, ensures reconstruction of crop stress indicators under interference.
- Security: not the primary focus, as typical farm data are generally of low sensitivity.
- Latency: optional, most events are not time-critical; exceptions for irrigation failures.
- Power efficiency: required, battery/solar field nodes and cameras must be energy efficient.
- Equipment requirements: dust, rain, UV-resistant housings; field-hardened connectors.
- Mobility Support: optional, mainly for drone flyovers (short burst uploads while airborne).

### 6.5. Railroad Monitoring

Critical linear infrastructures such as railways and pipelines extend through sparsely populated regions with limited communications coverage. Monitoring systems must operate autonomously for long periods and report anomalies such as vibration patterns, temperature deviations, or structural shifts. LoRa nodes placed at regular intervals enable reliable long-range communication with minimal power consumption. Incorporating basic visual confirmation, captured by periodic drone flights or stationary low-power cameras, further enhances the ability to detect and verify faults or security risks along these extended assets. Requirements for railroad monitoring:

- Data size & compression: can be implemented aggressive compression.
- Long range (coverage): required, linear assets extend through remote areas with sparse coverage.
- Image Integrity: required, complete and verifiable images are needed for safety-critical decisions.
- Security: not the primary focus, reliability and autonomy are prioritized, with stronger security measures recommended in high-risk regions.
- Latency: quicker alerts reduce safety risks and operational downtime.
- Power efficiency: required, remote nodes must run autonomously for extended periods.
- Equipment requirements: vibration-resistant, temperature-tolerant, EMI-aware installations.
- Mobility Support: optional, applies mainly to inspection drones or patrol vehicles.

### 6.6. Remote Medical Monitoring and Emergency Healthcare Support

Remote healthcare delivery often takes place in regions with limited or unreliable cellular or Wi-Fi connectivity, restricting the ability to transmit patient data from wearable devices or portable diagnostic tools. LPWAN technologies such as LoRa provide long-range, low-power communication suitable for connecting medical sensors and monitoring equipment across dispersed communities. By enabling the transfer of vital signs, emergency alerts, and small, compressed medical images, LoRa enhances clinical oversight and supports timely decision-making in environments where conventional communication infrastructure is unavailable or unstable. Requirements for Remote Medical Monitoring:

- Data size & compression: can be implemented aggressive compression.
- Long Range (coverage): recommended, rural/home settings benefit but may be near gateways.
- Image Integrity: required, diagnostic decisions rely on complete and verified images.
- Security: required, medical data must be protected and trustworthy for clinical use.
- Latency: emergency alerts need rapid response.
- Power efficiency: required, wearables and portable diagnostics must operate for long durations.
- Equipment requirements: medical-grade or robust field kits with reliable power backup.
- Mobility Support: recommended, patients may move within homes/communities; links must tolerate roaming.

### 6.7. Minimum Image Quality Requirements

Determining the minimum acceptable image quality is essential when evaluating the feasibility of image transmission over LoRa networks. Unlike conventional image communication systems, LoRa operates under severe constraints in terms of bandwidth, payload size, and transmission time. Consequently, image quality requirements vary significantly according to the application and the purpose of the transmitted image.

Image quality can be assessed using objective metrics such as the Structural Similarity Index (SSIM) [60] and the Learned Perceptual Image Patch Similarity (LPIPS) [61] metric. SSIM evaluates the preservation of structural information between the original and reconstructed image, whereas LPIPS measures perceptual similarity by estimating how similar two images appear to a human observer. Lower LPIPS values indicate better perceptual quality, while higher SSIM values indicate greater structural similarity.

In general, SSIM values above 0.80 indicate acceptable image reconstruction for monitoring and situational-awareness tasks, while values above 0.90 correspond to high-quality image reconstruction suitable for detailed visual inspection. For LPIPS, values below 0.30 typically indicate acceptable perceptual quality, whereas values below 0.15 represent high visual fidelity with minimal perceptual distortion.

However, acceptable quality levels depend strongly on the application. For example, wildlife monitoring and smart agriculture often require only the identification of animals, vegetation conditions, or environmental changes, allowing stronger compression and lower image quality. In contrast, applications such as railroad inspection, disaster assessment, and remote medical monitoring require higher image fidelity because operational decisions depend directly on the correct interpretation of visual information. Table 4 summarises representative image-quality requirements for selected LoRa-based image transmission applications. The image resolutions and SSIM and LPIPS thresholds presented in Table 4 represent illustrative design targets proposed by the authors for evaluating LoRa-based image transmission systems, rather than strict operational limits or universally established quality requirements.

## 7. Challenges and Approaches for Image Transmission over LoRa Networks

While LoRa has proven highly effective for transmitting low-bandwidth environmental data, such as temperature, humidity, or gas concentrations, its use in more data-intensive scenarios remains limited. Image transmission represents a particularly challenging application because images require substantially more data than conventional environmental measurements and therefore place greater demands on LoRa’s limited data rate and payload capacity. LoRa is widely regarded as unsuitable for high-bit-rate data transmission, such as images or audio, due to the narrow bandwidth of its physical layer, which limits the achievable data rate. This constraint makes it difficult to handle larger files or media formats that typically demand greater bandwidth. The study [62] highlights that LoRa is not suitable for visual IoT applications, where large amounts of data (like video) need to be transmitted. Instead, the use of 4G LTE mobile networks is emphasized as more appropriate for visual IoT devices due to the higher data bandwidth required for real-time video transmission. Nevertheless, despite LoRa’s inherent limitations in bandwidth and data rate, its long-range, low-power characteristics have prompted increasing research interest in adapting it for image transmission, particularly in scenarios where conventional high-bandwidth protocols are impractical due to energy constraints or lack of infrastructure. Accordingly, this section focuses specifically on image transmission over LoRa and synthesises the approaches proposed to address its principal limitations. The reviewed studies can be broadly grouped into four interconnected strategies: image-data reduction, packetisation and reliability, communication and network optimisation, and application-specific transmission strategies. The discussion compares their effects on image size, transmission time, reliability, energy consumption, coverage, image quality, and system complexity.

Early work demonstrated the feasibility of adapting LoRa for low-resolution visual sensing. Study [63] developed a low-cost, low-power image sensor using a Teensy 3.2 [64] and a LoRa module based on the Semtech SX1276 chip [65], with a Raspberry Pi [55] used as the receiving gateway. Firstly, raw image (128 × 128 pixels, 8-bit grayscale) was compressed using a JPEG-like encoding technique, which divided the image into 8 × 8 pixel blocks. To transmit the image, it was divided into packets up to 255 bytes each (including headers). The system used change detection to identify significant changes, the entire image is transmitted once a predefined change threshold is met. However, this approach can be challenging in outdoor environments with continuous changes in lighting and background movements.

Similarly, ref. [66] investigated image transmission using SX1276-based LoRa modules [65] and STM32L microcontrollers [67] connected to Raspberry Pi Zero devices [55]. Images with a resolution of 160 × 120 pixels were converted to grayscale, compressed using JPEG and JPEG 2000 [68], and divided into 220-byte packets. However, they highlighted challenges such as packet loss, where missing packets could prevent image reconstruction, making the fragmented approach vulnerable. It noted issues with compression, particularly JPEG’s quality degradation. Moreover, the study discussed trade-offs in transmission settings, where higher data rates reduced time but increased packet loss, emphasizing limitations in using LoRa’s low bandwidth for multimedia data transmission. Together, refs. [63,66] show that image compression alone does not guarantee reliable transmission. Although grayscale conversion and compression reduce the amount of data, packet loss can still prevent complete image reconstruction. The studies also indicate a trade-off between transmission time and reliability, with configurations providing higher data rates potentially reducing latency while increasing packet loss.

### 7.1. Image Compression and Data Reduction Techniques

Image transmission over LoRa networks presents significant challenges due to strict limitations in bandwidth, data rate, and payload size. To address these constraints, researchers have developed a range of novel techniques that adapt LoRa technology for visual data applications. The most direct way of reducing the communication burden is to reduce the amount of image data before transmission. Across the reviewed studies, this has been achieved through grayscale conversion, image resizing, JPEG/JPEG 2000, WebP, wavelet transformation, compressive sensing, and application-specific compression schemes. These approaches reduce transmission time and energy requirements but introduce different trade-offs in image quality, computational complexity, and reconstruction reliability.

Study [69] investigated image transmission for mangrove monitoring using Arduino-based nodes [53] with LoRa RN 2903 transceivers [70]. Images were captured, converted to hexadecimal representation, divided into packets, and transmitted between a skylift-mounted transmission node and a vehicle-mounted receiver. Image quality was evaluated using Peak Signal-to-Noise Ratio, Mean Squared Error (MSE), and Structural Similarity Index. This study demonstrated that LoRa technology can be used for transmitting images, despite its inherent limitations in bandwidth and data rate. Image data was converted into hexadecimal format and then split into smaller packets. This method was chosen because it fits within the transmission capacity allowed by the LoRa physical layer. The packets were sent sequentially, with pauses between transmissions. By splitting the data into smaller hexadecimal packets, the study could implement packet-based error handling. If a packet was lost or corrupted, only that specific packet needed to be retransmitted, rather than the entire image, thus optimizing the transmission process. However, the major drawback of encoding the data into hexadecimal is that each byte of original binary data are represented as two ASCII characters. This effectively doubles the size of the data being transmitted, since each hex character takes 8 bits. This leads to longer transmission times and can exacerbate the bandwidth limitations of LoRa. Also, Converting the image to hexadecimal and splitting it into packets could consume more processing power and energy, especially for low-power devices. While hexadecimal encoding can be useful for small data transmissions, it is less efficient for large data, such as high-resolution images, due to the increased data size and limited transmission rate of LoRa. Thus, while the method was suitable for the study’s specific use case, it may not be ideal for more data-intensive applications without further optimisation. This result demonstrates that data representation is itself an important design consideration. Although hexadecimal encoding facilitates packet handling and selective retransmission, it introduces substantial representation overhead compared with binary transmission. Therefore, the approach improves packet-level handling at the expense of transmission efficiency and energy consumption.

Fan and Ding [6] proposed a LoRa-based low-power wide-area protocol for visual sensor networks using a simplified star topology, camera-node registration, lightweight encryption, and baseline and continuous operating modes. Although the protocol addresses power efficiency and visual-data delivery, the absence of real-world tests or simulations limits the extent to which its performance can be quantitatively compared with the other approaches.

Another study [71] aimed to enhance long-range data transmission in LoRa networks by proposing a Multi-Access Mechanism adapted to LoRa’s physical layer, addressing the challenge of transmitting large data volumes such as images. By leveraging the Channel Activity Detection feature in LoRa chips, the mechanism could detect when the channel was free for transmission, thereby reducing collisions. If the channel was busy, the node would enter a Deep Sleep Mode to conserve energy before rechecking channel availability, repeating this process until successful transmission. This adaptive Carrier Sense Multiple Access approach demonstrated reduced energy consumption and improved transmission efficiency in dense IoT networks. The study successfully transmitted compressed images using LoRa by implementing a custom compression scheme optimized for low-resource devices and long-range communication. The images, captured by a Teensy32 board [64] with a uCamII camera, were compressed to sizes between 900 and 1200 bytes, then divided into smaller packets for transmission. The Carrier Sense Multiple Access mechanism, combined with adjustable quality factor settings for image compression, helped avoid packet collisions and ensured robust image transmission, even with some packet losses. While the approach proved effective in real-world scenarios, there are challenges such as Channel Activity Detection reliability over long distances and increased latency.

Chen and colleagues [72] combined image reduction with packet-level and channel-level optimisation. Images captured using a Brinno TLC200 Pro [73] at 3280 × 2464 pixels were resized to 480 × 320 pixels and compressed to as low as 9 KB using the Pillow Python library [74]. The proposed multi-Packet LoRa protocol used batch transmission and bit-vector acknowledgements to selectively retransmit lost packets, while a data-channel reservation mechanism reduced congestion in multi-node environments. The study primarily focused on improving image transmission efficiency over LoRa networks using the multi-Packet LoRa protocol, the authors indicated the limitations of single-hop transmissions in real-world environments. So, the authors implied that enhancing the multi-Packet LoRa protocol for multi-hop networks could facilitate more efficient data transmission. This study demonstrates the benefit of combining compression with selective retransmission and channel management. However, it also indicates that single-hop operation can limit scalability and coverage in real-world deployments.

Overall, the compression studies show that reducing image size is the most consistently adopted strategy for making image transmission feasible. However, the studies do not identify a universally optimal compression method. Lightweight methods are more appropriate for constrained nodes, whereas advanced methods can achieve greater data reduction but require additional processing. Furthermore, compression must be evaluated jointly with image quality because reducing transmission time at the expense of excessive visual degradation may not satisfy the requirements of the target application.

### 7.2. Packetisation and Reliability for Image Transmission

Because compressed images can still exceed the payload supported by a single LoRa packet, packetisation is a fundamental requirement for image transmission. The reviewed studies employ sequential packet transmission, packet identifiers, acknowledgements, selective retransmission, and progressive delivery to address packet loss and image reconstruction. The main trade-off is between reliability and transmission overhead: retransmission improves image integrity but increases latency and energy consumption. The images were divided into packets up to 255 bytes each (including headers) in [63], while [66] split images into packets of 220 bytes. These approaches demonstrate why packet-level recovery is important: losing a single packet can prevent successful image reconstruction, whereas selective recovery allows only the missing or corrupted packet to be retransmitted.

Chen et al. [72] extended this concept through a bit-vector acknowledgement mechanism, allowing the receiver to indicate which packets were successfully received and which required retransmission. Similarly, ref. [75] employed acknowledgement-based retransmission in a single-hop network. These approaches are more efficient than retransmitting an entire image, but the additional control traffic increases transmission overhead and latency.

Therefore, packet reliability should be considered together with image compression. A smaller compressed image requires fewer packets and consequently reduces the probability of packet loss and the amount of retransmission required. This creates an important cross-layer relationship between image compression and communication reliability that is not fully captured when these techniques are evaluated independently.

### 7.3. Communication and Network Optimisation

Transmitting images over LoRa in practical settings requires approaches that mitigate the limitations of low bandwidth, constrained data rates, and small payload sizes. Beyond reducing image size, several studies optimise the way packets are transmitted through the LoRa network. These approaches include adaptive channel access, channel reservation, SF/BW optimisation, parallel transmission, cooperative transmission, and multi-hop routing. Their primary objectives are to reduce latency, avoid collisions, improve energy efficiency, or extend coverage.

For example, ref. [71] used Channel Activity Detection and Carrier Sense Multiple Access to reduce collisions and conserve energy, while ref. [72] used data-channel reservation to manage congestion. These approaches are particularly relevant in multi-node networks, where simultaneous packet transmissions can significantly reduce packet delivery performance. However, channel sensing and reservation introduce additional waiting time and control overhead.

In contrast, ref. [76] addressed transmission latency through parallel delivery. A compressed image was divided into packets and distributed among multiple Raspberry Pi 3 B+ [55] nodes operating with different SFs. MQTT coordinated packet distribution, while the receiver reassembled the image. Based on the study, parallel transmission offered faster transmission, as it sends multiple packets simultaneously across different channels, reducing total transmission time compared to sequential transmission, which sends packets one after another. Also, parallel transmission enhances reliability, as data are spread across multiple channels, allowing uninterrupted flow even if one channel encounters interference. However, parallel transmission demands more complex hardware setups, increasing system costs and power consumption, which may not be ideal for low-power applications. In contrast, sequential transmission is simpler, requires less hardware, and is often more energy-efficient, making it preferable for low-data or low-power applications. Thus, parallel transmission should not be considered universally superior to sequential transmission. It is advantageous when transmission latency is critical, whereas sequential transmission remains attractive for low-power applications where hardware simplicity and energy consumption are more important.

Multi-hop transmission provides another means of addressing coverage limitations. Kim and colleagues [77] used Raspberry Pi 4 devices [55] and Adafruit RFM95 LoRa modules [78] to relay image packets through intermediate nodes. The study demonstrated reliable transmission for a single sender but identified scalability and collision issues when multiple senders shared a relay. Therefore, multi-hop can extend coverage without requiring additional gateways, but each relay introduces additional forwarding overhead and potential collision points.

Similarly, ref. [79] investigated cooperative and parallel transmission in which multimedia data were divided into chunks and distributed among ESP32-S3 devices [54] equipped with SX1276 modules [65]. A water-filling algorithm [80] allocated chunks according to battery life, SF, availability, and previous transmission success, while time-slotting reduced collisions. The approach reduced data collection time compared with sequential transmission but required multiple LoRa modules at the gateway and increased coordination, hardware requirements, and cumulative energy consumption.

Overall, communication-level approaches reveal a consistent trade-off. Increasing parallelism can reduce latency, multi-hop can extend coverage, and adaptive access can reduce collisions, but these improvements require additional hardware, coordination, energy, or protocol complexity. Consequently, the appropriate communication architecture depends on whether the application prioritises latency, coverage, energy efficiency, or simplicity.

### 7.4. Application-Specific and Advanced Image Transmission Strategies

The effectiveness of the above techniques is strongly dependent on the application. The literature shows that an approach that performs well in a stable monitoring environment may be unsuitable for dynamic surveillance, while a method that minimises latency may require more energy and hardware than a periodic sensing application can justify.

To monitor agricultural environments [81] proposes a method that minimised the data sent over the network. Instead of transmitting complete images continuously, which would consume too much data, the system breaks each image into small segments or “patches.” Only the patches that show a meaningful change since the last image are transmitted, as most parts of the scene in agricultural areas remain unchanged over time. Performance testing showed that 24% of cycles required transmission, significantly cutting data needs and maintaining 95% image similarity. The study used a Raspberry Pi 3 Model B with a Pi Camera 2 [55] and Arduino Uno with a LoRa Shield [53] as the end node to capture images and detect changes, using LoRa for communication. A gateway forwards data to an AWS EC2 instance [82] that acts as a back-end server, reconstructing images for near real-time web access. The camera captures a grayscale, low-resolution (160 × 160 pixels) image of the monitored area at regular intervals. The mage is divided into small, fixed-size grid patches (e.g., 10 × 10 pixels) to isolate specific sections for easier change detection. However, the patch-based LoRa transmission method is not suitable for constantly changing environments, as it relies on stable backgrounds with infrequent changes. In dynamic settings, frequent small changes would trigger continual data transmissions, overwhelming LoRa’s limited bandwidth and negating the method’s efficiency. This approach works best in stable environments, where only significant changes need to be monitored and transmitted. Therefore, ref. [81] demonstrates that application-level redundancy reduction can be more effective than attempting to increase the communication rate. However, the approach is highly dependent on scene stability and is less suitable for dynamic environments.

The study [83] focused on comparing the efficiency of transmitting images over LoRa by evaluating two encoding methods—JPEG [68] and Webp with Base64 encoding [84]—to identify the most effective compression approach. Key metrics used for comparison included Peak Signal-to-Noise Ratio, Structural Similarity Index, and transmission time. The authors conducted transmission tests over a 1.5-km range using Raspberry Pi 3 B+ devices [55] equipped with SX1276 LoRa chips [65]. In these tests, a 200 × 150 image compressed with JPEG reduced to 20.03 KB, requiring 47.7 s to transmit, while Webp + Base64 reduced it to 5.51 KB and 25.7 s. Both methods provided similar image quality, but Webp + Base64 demonstrated significantly faster transmission. The study concludes that Webp + Base64 is a viable method for reducing transmission time in IoT applications that require image data. This direct comparison provides evidence that encoding efficiency can substantially affect transmission time. Importantly, however, image size should be considered alongside image quality and encoding overhead rather than as the only performance indicator.

Similarly, ref. [13] demonstrated the potential of aggressive image reduction through wavelet transformation and compressive sensing, reducing a 128 × 128-pixel image to approximately 5% of its original size and transmitting it in 2.51 s using four packets. However, the compressive sensing and wavelet transformation techniques used for compression add computational complexity, which may not be ideal for battery-powered, low-processing-capacity IoT devices. Moreover, the experiment relies on National Instruments NI-2920 Software-Defined Radios [85] to configure LoRa parameters with high flexibility, are relatively costly and complex compared to standard commercial LoRa modules, making the setup less feasible for large-scale or low-cost IoT deployments. Thus, ref. [13] represents a trade-off between communication efficiency and implementation complexity: greater compression can significantly reduce the communication burden, but the associated processing and hardware requirements may limit deployment on highly constrained nodes.

A study by Guerra and colleagues [86] proposed an image encoder where they transformed RGB images [87] to the YCoCg colour model [88]. The encoding process included several key steps. They first transformed the images to the YCoCg colour model, separating brightness from colour and allowing aggressive compression of the colour components through subsampling. Next, they applied wavelet decomposition, which split the image into low- and high-frequency sub-bands. By prioritizing low-frequency components that hold essential image structure and discarding some high-frequency details. The compressed data was then quantized and encoded using Huffman coding [89] to reduce redundancy, followed by packetization for transmission. Given LoRa’s 146-byte packet size limit, the encoder divided the compressed data into small packets, each with metadata for accurate reconstruction. The packets were sent in a progressive order: lower-resolution, low-frequency sub-bands were transmitted first, allowing the receiver to display a low-quality preview. The researchers used Raspberry Pi 3B devices [55] connected to SX1272 LoRa chips [90] for both sending and receiving the packets. Transmission tests were conducted at distances of 2068 m, 4848 m, and 16,150 m to evaluate the approach over varying ranges. The results showed that the method achieved a reduction in transmission time compared to uncompressed images, making it energy-efficient for remote, battery-powered applications. However, packet loss could impact image reconstruction, and the predefined Huffman [89] codebooks may not optimize compression for diverse image types. This approach demonstrates the value of progressive transmission because the receiver can obtain a lower-quality preview before the complete image is received. However, packet loss can affect reconstruction, and predefined Huffman codebooks may not provide optimal compression for different image types.

Another study [91] compared two methods, Base64 encoding [92] and JPEG compression [68], to improve LoRa-based image transmission. Base64 encoding achieves a high packet reception rate but is vulnerable to decoding errors under poor signal conditions. JPEG compression, using both lossy and lossless methods, enables faster transmission of smaller images and shows greater resilience to errors despite a slightly lower PRR than Base64. The study’s experimental setup involved a LoRa network using Raspberry Pi devices [55] and Sx1276 modules [65], configured to measure transmission time, Signal-to-Noise Ratio, and Bit Error Rate across different image sizes and parameters. Results indicate that JPEG compression is more efficient for smaller images, reducing transmission time while retaining image quality, though some colour alterations were noted for larger images. This comparison further demonstrates that Packet Reception Rate alone does not adequately represent image-transmission performance. Image size, transmission time, reconstruction success, and image quality should be considered together when evaluating alternative encoding schemes.

The study [7] aimed to create an energy-efficient system for automating the reading of traditional utility meters by transmitting images of meter counters using LoRa. This approach eliminates the need for expensive replacement of analog meters with smart meters. To achieve this, the authors proposed a LoRa multi-packet protocol designed for image transmission. By dividing images into smaller packets, the protocol facilitates efficient data transfer over long distances with minimal power consumption, demonstrating a novel application of LoRa technology beyond its conventional use for small data payloads. The system was evaluated for performance based on transmission time, image quality, and energy consumption, demonstrating its potential for real-world IoT applications. The experiment used a combination of hardware, including an OV7670-FIFO camera [93] for image capture, an EBYTE E220-900T22D LoRa module [94] for data transmission, and Arduino microcontrollers, MEGA for transmission and UNO for reception [53]. A 9 V, 3000 mAh battery powered the system, and tests were conducted over varying distances (10 m to 1 km). Experimental results showed successful transmission of images up to 1 km, with acceptable quality (low Mean-Square Error and high Peak Signal-to-Noise Ratio) at shorter distances, with excellent image quality up to 250 m. However, beyond 500 m, packet loss began to degrade image quality. Transmission speed varied from 9 s at 62.5 kbps for close distances to 25 s at lower speeds for 1 km. Energy analysis estimated a battery life of 2.11 months for periodic transmissions. The results demonstrate that periodic meter-reading is a suitable application because long transmission times can be tolerated when images are captured infrequently. However, packet loss beyond 500 m degraded image quality, demonstrating the dependence of image quality on communication reliability.

The findings from [7,13,81,83,86] demonstrate that the optimal image-transmission strategy depends strongly on the application. Stable agricultural scenes benefit from patch-based transmission, compression-focused approaches can reduce transmission time for general image monitoring, progressive encoding can provide useful information before complete reconstruction, and periodic meter reading can tolerate relatively long transmission times. These results reinforce that LoRa should primarily be considered for low-frequency, low-resolution, or highly compressed image applications rather than continuous high-resolution visual transmission.

### 7.5. Comparative Analysis of LoRa-Based Image Transmission Approaches

Table 5 consolidates the principal experimental characteristics and performance indicators reported in the literature.

The comparative analysis identifies major patterns across the reviewed literature. First, data reduction is the most consistently used strategy for overcoming LoRa’s limited bandwidth. Grayscale conversion, resizing, JPEG/JPEG2000, WebP, wavelet transformation, compressive sensing, and selective patch transmission all reduce the amount of information that must be transmitted [13,63,66,71,72,81,83,86,91]. However, greater compression does not necessarily provide a universally better solution because aggressive or computationally complex compression may increase processing requirements or reduce image quality.

Packetisation and reliability mechanisms have evolved from simple image fragmentation toward selective packet recovery. Studies [63,66,72,75] demonstrate the importance of packet-level error handling because the loss of individual packets can prevent successful image reconstruction. Acknowledgements and selective retransmission improve reliability by recovering only missing packets, but they introduce additional control traffic, latency, and energy consumption.

Application characteristics strongly determine which strategy is most appropriate. Patch-based transmission is effective when the monitored scene is stable [81], while periodic meter-reading applications can tolerate relatively long transmission times [7]. In contrast, continuously changing surveillance environments are less suitable for change-based transmission, and real-time video remains impractical for conventional LoRa because of its fundamental data-rate limitations [62].

The comparative analysis also highlights a broader research gap: future LoRa image-transmission systems should be evaluated using a unified set of metrics, including image size and compression ratio, LoRa parameters, transmission distance, latency, energy consumption, packet delivery ratio. Such evaluation would enable meaningful comparison between competing approaches and would provide clearer evidence regarding which techniques are most suitable for specific real-world applications.

## 8. Overview of Equipment and Reviewed Works

Across the reviewed literature, several hardware components are frequently used in the design of LoRa-enabled IoT systems for environmental monitoring. The most commonly employed microcontrollers include the Arduino [53], ESP32 [54], and Raspberry Pi [55], valued for their open-source, low power consumption, and ease of integration. For LoRa communication, the SX1276/78 [65], ref. [95] modules are widely adopted due to their long-range capabilities and broad compatibility. These hardware choices reflect a preference for low-cost, energy-efficient, and programmable platforms suited to remote or resource-constrained environments. A detailed list of the reviewed studies and their respective applications is provided in Table 6.

## 9. Future Directions for Image Transmission over LoRa

The optimisation of image transmission over LoRa networks remains both challenges and opportunities, particularly in addressing constraints such as limited bandwidth, low power, and the need for high reliability. LoRa’s potential for low-power, long-range communication makes it an attractive solution for various IoT applications, but efficient handling of image data requires further development. Based on a review of existing research, several future directions emerge to overcome these limitations and enhance the feasibility of LoRa for image-based applications.

One key challenge is LoRa’s limited bandwidth and payload capacity, which restrict the amount of data that can be transmitted efficiently. Addressing this issue involves implementing advanced compression techniques that reduce image size without compromising essential details. For instance, content-aware compression can prioritise regions of interest while compressing non-critical areas more aggressively. Progressive transmission methods, where low-resolution previews are sent first followed by additional detail layers, offer another solution to manage bandwidth constraints effectively. These approaches are worth pursuing because they directly tackle one of LoRa’s most significant limitations, enabling faster transmission and conserving energy in resource-constrained environments.

Packet loss remains a persistent issue, especially in multi-hop networks where data travels through several relay nodes. Forward Error Correction methods can add redundancy to packets, enabling the reconstruction of lost data without retransmission. Additionally, adapting acknowledgment systems to multi-hop setups can improve reliability, ensuring that errors are detected and corrected across the entire transmission path. These strategies are particularly valuable in remote or lossy environments, where retransmissions are expensive and energy-intensive, making them essential for enhancing LoRa’s reliability.

Energy efficiency is another critical concern for LoRa-based IoT devices, which are often battery-powered and deployed in remote locations. Techniques such as adaptive duty cycles and optimised power control can help conserve energy. Integrating energy-harvesting technologies like solar panels could further extend device lifetimes. These measures are worth implementing because they address the practical constraints of IoT devices, allowing networks to operate for extended periods without frequent maintenance.

Scalability poses challenges in dense networks with high device counts or large data volumes. Cooperative transmission techniques, where groups of devices share the problem of transmitting data, can reduce blockage and improve data flow. Similarly, optimising gateway designs to handle higher packet arrival rates through parallel processing and better buffer management can increase the capacity of LoRa networks. These efforts are critical as scalability is essential for expanding IoT deployments in urban and industrial environments.

Data security and privacy must be prioritised, particularly in sensitive applications such as healthcare and surveillance. To ensure efficient processing within LoRa’s constrained bandwidth and computational capabilities, compression should be applied before encryption. This preserves compression efficiency, as encrypted data are inherently random and resistant to further size reduction. Lightweight encryption schemes, such as AES-128, along with packet authentication mechanisms, can then be used to secure transmissions without overwhelming system resources. However, care must be taken to guard against potential side-channel attacks that exploit patterns in compressed-and-encrypted data. Additionally, compressed data can be more sensitive to transmission errors, so robust error detection and correction mechanisms are essential. Proper implementation of these measures helps maintain data integrity and confidentiality, enhancing the trustworthiness of LoRa-based systems

ML offers transformative potential to address many of these challenges, enabling LoRa networks to adapt intelligently to varying conditions. For instance, ML can optimise compression strategies by dynamically adjusting compression levels based on image content and network conditions. Lightweight models, such as convolutional neural networks, can identify regions of interest and apply tailored compression, balancing data size with quality. Autoencoders, designed for efficient representation learning, can further enhance this process by compressing images into compact latent vectors while preserving essential features. This approach is particularly valuable for applications requiring the preservation of critical details, such as remote diagnostics or surveillance.

Network congestion and collisions, common in dense IoT environments, can be mitigated using predictive ML models. Algorithms like Long Short-Term Memory networks can forecast traffic patterns and adapt transmission schedules to avoid conflicts. Reinforcement learning can dynamically adjust parameters such as SF and transmission power to optimise network performance. These predictive and adaptive capabilities significantly enhance LoRa’s efficiency and are worth pursuing in environments prone to congestion.

Packet loss and corruption can also benefit from ML-based solutions. Models like Generative Adversarial Networks can reconstruct missing or corrupted data by learning patterns in image content, reducing the need for retransmissions. Such methods are especially useful in lossy environments, where reliable data delivery is challenging. Additionally, ML can aid in resource allocation by predicting optimal settings for parameters such as power levels and transmission intervals, ensuring balanced energy use across devices while maintaining throughput.

For real-time image transmission, ML can play a crucial role by prioritising and transmitting critical frames or incremental updates. Edge AI can preprocess and prioritize data for immediate needs, while cloud systems handle more advanced tasks such as pattern recognition or anomaly detection. This combination ensures that real-time demands are met efficiently, making it particularly valuable for time-sensitive applications like safety monitoring and environmental surveillance.

Addressing the challenges of LoRa image transmission requires a combination of traditional optimization methods and ML-driven innovations. Non-ML approaches focus on improving compression, reliability, energy efficiency, and scalability, which form the foundation for efficient LoRa networks. Meanwhile, ML enables advanced adaptation and prediction capabilities, allowing networks to respond dynamically to complex, real-world conditions. Both approaches are complementary and essential for the continued development of LoRa as a robust solution for image-rich IoT applications.

## 10. Conclusions

LoRa technology has emerged as a foundational enabler for a wide range of IoT applications, particularly in environmental monitoring. Its ability to support long-range, low-power communication makes it ideal for deployments in remote, infrastructure-limited, or resource-constrained settings. Across various domains, such as water quality assessment, air pollution tracking, radiation detection, wildlife monitoring, and underground mining, researchers have demonstrated LoRa’s effectiveness in enabling real-time, scalable, and cost-efficient sensing systems. These implementations often leverage open-source hardware, renewable energy sources, and intelligent algorithms to enhance system autonomy and adaptability.

The versatility of LoRa is further illustrated by its successful integration into diverse environmental contexts, from the Amazon River basin to smart grid infrastructure. Innovations such as mesh networking protocols, dual-radio platforms, and machine learning-enhanced communication strategies have expanded LoRa’s capabilities beyond traditional star-topology networks. These advancements not only improve coverage and reliability but also open new possibilities for deploying LoRa in dynamic, mobile, or harsh environments.

Despite its strengths, LoRa is generally considered unsuitable for transmitting high-bit-rate data, such as images or audio, because the narrow bandwidth of its physical layer limits the achievable data rate. This constraint makes it challenging to handle larger files or media formats that typically require more bandwidth. However, researchers have developed several strategies to overcome these limitations. By using methods like compressing data, creating better ways to send messages, and using multi-hop systems (where messages are passed from one device to another), they have found ways to make LoRa useful even for tasks like sending pictures or videos.

This study shows how LoRa can handle even challenging tasks, like transmitting large image files, by overcoming common problems such as losing packets, using too much energy, or ensuring the data arrives correctly. These advancements open up new possibilities for using LoRa in more complex systems.

Future research should focus on ML-driven adaptive compression, lightweight encryption, energy-efficient transmission scheduling, and the seamless integration of LoRa with edge-computing platforms for real-time processing.

By addressing these issues and continuing to improve, LoRa has the potential to become a flexible and cost-effective technology for many IoT uses. Whether it’s for tracking environmental changes, monitoring agriculture, or even sending pictures from remote locations, LoRa can support a wide variety of applications, making the IoT more accessible and affordable for everyone.

## Figures and Tables

**Table 1:** Comparison of wireless communication technologies relevant to IoT applications.

Protocol	Frequency	Typical Range	Data Rate	Topology	Power Efficiency	Typical Applications
Bluetooth/BLE	2.4 GHz ISM	~10–100 m, depending on configuration	Up to ~2 Mbps for BLE	Star; mesh supported by Bluetooth Mesh	High for BLE applications	Wearables, local sensing, consumer IoT
Zigbee	2.4 GHz; sub-GHz in some regions	~10–100 m per hop; extended through mesh	Up to 250 kbps	Mesh	High	Smart homes, lighting, building automation
Z-Wave	Regional sub-GHz bands	~30–100 m per hop; mesh extension	Up to 100 kbps	Mesh	High	Smart locks, security, HVAC, home automation
Sigfox	Sub-GHz ISM	Several kilometres; potentially tens of kilometres in favourable rural conditions	Up to ~100 bps uplink	Star	Very high for small, infrequent transmissions	Asset tracking, agriculture, remote monitoring
NB-IoT	Licensed cellular spectrum	Typically kilometres; dependent on network deployment	Up to hundreds of kbps under suitable conditions	Cellular/star	High with PSM/eDRX	Smart metering, utilities, smart cities, industrial IoT

**Table 2:** LoRa Parameters Examples.

SF	BW (kHz)	CR (4/x)	Symbol Duration (ms)	Data Rate (bps)
7	125.0	4/5	1.024	5468.75
7	125.0	4/6	1.024	4557.29
7	125.0	4/7	1.024	3906.25
7	125.0	4/8	1.024	3417.97
8	125.0	4/5	2.048	3125.0
8	125.0	4/6	2.048	2604.17
8	125.0	4/7	2.048	2232.14
8	125.0	4/8	2.048	1953.12
9	125.0	4/5	4.096	1757.81
9	125.0	4/6	4.096	1464.84
9	125.0	4/7	4.096	1255.58
9	125.0	4/8	4.096	1098.63
10	125.0	4/5	8.192	976.56
10	125.0	4/6	8.192	813.8
10	125.0	4/7	8.192	697.54
10	125.0	4/8	8.192	610.35
11	125.0	4/5	16.384	537.11
11	125.0	4/6	16.384	447.59
11	125.0	4/7	16.384	383.65
11	125.0	4/8	16.384	335.69
12	125.0	4/5	32.768	292.97
12	125.0	4/6	32.768	244.14
12	125.0	4/7	32.768	209.26
12	125.0	4/8	32.768	183.11
7	250.0	4/5	0.512	10,937.5
7	250.0	4/6	0.512	9114.58
7	250.0	4/7	0.512	7812.5
7	250.0	4/8	0.512	6835.94
8	250.0	4/5	1.024	6250.0
8	250.0	4/6	1.024	5208.33
8	250.0	4/7	1.024	4464.29
8	250.0	4/8	1.024	3906.25
9	250.0	4/5	2.048	3515.62
9	250.0	4/6	2.048	2929.69
9	250.0	4/7	2.048	2511.16
9	250.0	4/8	2.048	2197.27
10	250.0	4/5	4.096	1953.12
10	250.0	4/6	4.096	1627.6
10	250.0	4/7	4.096	1395.09
10	250.0	4/8	4.096	1220.7
11	250.0	4/5	8.192	1074.22
11	250.0	4/6	8.192	895.18
11	250.0	4/7	8.192	767.3
11	250.0	4/8	8.192	671.39
12	250.0	4/5	16.384	585.94
12	250.0	4/6	16.384	488.28
12	250.0	4/7	16.384	418.53
12	250.0	4/8	16.384	366.21

**Table 3:** Frequency bands and their duty cycle.

Band	Operational Frequency Band	Duty Cycle Limits
H	433.05–434.79 MHz	10%
K	863.00–865.00 MHz	0.1%
L	865.00–868.00 MHz	1%
M	868.00–868.60 MHz	1%
N	868.70–869.20 MHz	0.1%
P	869.40–869.65 MHz	10%
Q	869.70–870.00 MHz	1%

**Table 4:** Image-Quality Requirements for LoRa Transmission Applications.

Application	Typical Resolution	Minimum SSIM	Maximum LPIPS	Quality Priority
Mountain Search and Rescue	320 × 240 to 640 × 480	0.85	0.25	High
Disaster Relief Communications	320 × 240 to 640 × 480	0.85	0.25	High
Wildlife Monitoring	160 × 160 to 320 × 240	0.80	0.30	Moderate
Smart Agriculture	160 × 160 to 320 × 240	0.80	0.30	Moderate
Railroad Monitoring	320 × 240 to 640 × 480	0.90	0.15	Very High
Remote Medical Monitoring	320 × 240 to 640 × 480	0.90	0.15	Very High

**Table 5:** Summary of Image Specifications and Compression Methods.

Reference	Image Size/Resolution	Compression/Encoding
[7]	8 KB image	Multi-packet protocol/compressed format
[8]	VGA-quality image	Uncompressed; compression suggested
[63]	128 × 128, 8-bit grayscale	JPEG-like encoding
[66]	160 × 120	Grayscale, JPEG/JPEG2000
[69]	Image data	Hexadecimal encoding
[71]	900–1200 B compressed	Custom compression
[72]	480 × 320, as low as 9 KB	Resizing + Pillow compression
[81]	160 × 160 grayscale	Patch-based transmission
[76]	Compressed image	Compression + packetisation
[83]	200 × 150	JPEG/WebP + Base64
[77]	Image divided into packets	Packet segmentation
[75]	Resized image	Resizing + retransmission
[13]	128 × 128	Wavelet + compressive sensing
[86]	RGB images	YCoCg + wavelet + Huffman
[91]	Multiple image sizes	Base64/JPEG
[79]	Large multimedia file	No image compression; chunking

**Table 6:** Reviewed LoRa studies.

N	Authors and Year	Application	Technology Used	Key Features	Notable Outcomes
1	Pham, (2016) [63]	Visual surveillance	LoRa (SX1276), Teensy 3.2	JPEG-like compression	128 × 128 grayscale
2	Kirichek et al., (2017) [66]	Multimedia transmission	LoRa (SX1276), STM32L	JPEG/JPEG2000	Packet loss issues
3	Murata et al., (2018) [62]	Visual IoT feasibility	LoRa, 4G LTE	Bandwidth comparison	LoRa unsuitable for video
4	Jebril et al., (2018) [69]	Mangrove monitoring	LoRa (RN2903), Arduino	Hexadecimal encoding	Doubled data size
5	Fan and Ding, (2018) [6]	Video surveillance	LoRa	Star topology	No real-world tests
6	Pham (2018) [71]	Image transmission	LoRa (SX1276), Teensy 32	CSMA, Deep Sleep Mode	900–1200 byte images
7	Azmi Ali and Abdul Latiff, (2019) [51]	Environmental monitoring (island)	LoRa (SX1276), Arduino, Yagi antennas	SF11 vs. SF12 comparison	SF11: 98.18% PDR
8	Chen, Eager and Makaroff, (2019) [72]	Agricultural IoT	LoRa (SX1276), Raspberry Pi	Multi-packet protocol	9 KB images
9	Ji et al., (2019) [81]	Agricultural monitoring	Arduino with LoRa shield, Raspberry Pi	Patch-based detection	95% image similarity
10	Wei, Chen and Su (2019) [76]	Parallel image transmission	LoRa (SX1276), Raspberry Pi	SF diversity	Faster than sequential
11	Wei, Su and Chen (2020) [83]	Image encoding comparison	LoRa (SX1276), Raspberry Pi	JPEG vs. Webp + Base64	Webp faster
12	Chen et al. (2021) [39]	Water quality monitoring	LoRa (SX1278), Arduino UNO	4-layer architecture, cloud-based visualisation	<1% error, 100% success rate within 1.6 km
13	Manzano et al. (2021) [44]	Radiation monitoring	LoRaWAN, ATSAML21G18B	MQTT, ChirpStack	3.7 years battery life
14	Juliando et al. (2021) [8]	Environmental monitoring	LoRa Ra-02 (SX1278), Raspberry Pi	SF/BW tuning	5 km range
15	Sharma and Arya (2022) [46]	Air quality via UAV	LoRa, Raspberry Pi, UAV	Mobile gateways, cloud analytics	20 km range, pollutant forecasting
16	Kim et al. (2022) [77]	Multi-hop image transmission	LoRa (RFM95), Raspberry Pi	Concurrent senders	Improved range
17	Chaparro B., Pérez and Mendez (2022) [13]	Visual data compression	LoRa, SDR-Radio	Wavelet + CS	2.51 s for 128 × 128
18	Promput, Maithomklang and Panya-isara (2023) [40]	Water quality (robotic boat)	LoRa, ESP32	Mobile platform	95.5% accuracy, 2 km range
19	Kumar et al., (2023) [43]	Indoor air quality	LoRa (SX1278), ESP32	ML-based classification	MSE: 1.42 × 10^−4^
20	Wu and Liebeherr (2023) [48]	Mesh networking	LoRa (SX1276), ATmega328P	CottonCandy protocol	>90% PDR
21	Ray Chowdhury, Pramanik and Roy (2023) [45]	Underground coal mines	LoRa (SX1276), Arduino	Safety index algorithm	69.5% better coverage than WiFi
22	Sendra et al., (2023) [41]	Coastal/river water quality	LoRa (SX1278), Arduino, ESP32	TTN integration	910 m range (Arduino)
23	Guerra et al., (2023) [86]	Image encoding	LoRa (SX1272), Raspberry Pi	YCoCg, Huffman	Progressive preview
24	Jabbar et al., (2024) [1]	Rural water quality	LoRaWAN, Arduino	Solar-powered, TTN	13+ hrs operation
25	Hagh et al., (2024) [50]	Coastal soil pressure	LoRaWAN, Adafruit Feather M0	Delta encoding	0.5 mbar sensitivity
26	Naik, Reddy and Raj, (2024) [5]	Underground mining	LoRa (SX1276), ESP32	ThingSpeak integration	200 m tunnel range
27	Mohd Shari et al. (2024) [2]	Air quality (community)	LoRa Cytron RFM, Arduino	Telegram Bot alerts	Reliable performance
28	Kim, Kwak and Jenkins (2024) [75]	Single-hop reliability	LoRa (RFM95), Raspberry Pi	Resizing, ACK	Reduced packet loss
29	Dede, Jalajamony and Fernandez (2024) [91]	Image transmission	LoRa (SX1276), Raspberry Pi	Base64 vs. JPEG	JPEG faster
30	Torres, Nunez and Mauricio Villanueva (2024) [7]	Utility meter imaging	LoRa (E220), Arduino	Multi-packet protocol	2.11 months battery
31	Nurbay et al. (2024) [79]	Parallel image transmission	LoRa (SX1276), ESP32	Chunking, time slots	Faster transfer
32	Li et al. (2025) [47]	Emergency monitoring via UAV	LoRa (SX1262), STC15W4K56S4	UAV gateway, LTE/5G	17.25 km range
33	Ragnoli et al. (2025) [3]	Karst cave monitoring	LoRa (SX1262), NRF52	Asynchronous mesh	1-year battery life
34	Wang, Zhao and Niu (2025) [96]	Smart grid monitoring	LoRa (SX1276)	PSO & BPNN	25 km coverage
35	Rodrigues et al. (2025) [49]	Amazon river logistics	HTLRBL32L	Peer-to-peer + LoRaWAN	16.9 km range
36	Dao et al. (2025) [42]	Water level (battery-less)	LoRa (S76S), Ω–antenna	Solar + supercapacitor	RMSE: 2.5 mm
37	López-Escobar et al. (2025) [4]	Wildlife tracking	ASR6501 system	ML-based localisation	90–125 m error
38	Ahmad et al., (2025) [52]	Beehive monitoring	LoRa (SX1278), ESP32	BME680 + sound sensor	58 m range

## Data Availability

No new data were created or analyzed in this study. Data sharing is not applicable to this article.

## Abbreviations

The following abbreviations are used in this manuscript:

IoT	Internet of Things
LPWANs	Low-Power Wide-Area Networks
ML	machine learning
NB-IoT	Narrowband-IoT
LTE	Long-Term Evolution
CSS	Chirp Spread Spectrum
M2M	Machine-to-Machine
SF	Spreading factor
CR	Code Rate
FEC	Forward Error Correction
CRC	Cyclic Redundancy Check
UAV	Unmanned Aerial Vehicle
SSIM	Structural Similarity Index
LPIPS	Learned Perceptual Image Patch Similarity
